# Giant Response and Selectivity of Hansen Solubility Parameters-Based Graphene-SBS Co-Polymer Matrix Composite Room Temperature Sensor to Organic Vapours

**DOI:** 10.3390/polym16030309

**Published:** 2024-01-23

**Authors:** Rostislav Slobodian, Robert Olejnik, David John Dmonte, Jakub Sevcik, Jiri Matyas, Marek Jurca, R. Blessy Pricilla, Barbora Hanulikova, Petr Slobodian, Ivo Kuritka

**Affiliations:** 1 Centre of Polymer Systems, University Institute, Tomas Bata University in Zlín, Trida Tomase Bati 5678, 760 01 Zlín, Czech Republic; olejnik@utb.cz (R.O.); dmonte@utb.cz (D.J.D.); j4sevcik@utb.cz (J.S.); matyas@utb.cz (J.M.); jurca@utb.cz (M.J.); pricilla@utb.cz (R.B.P.); hanulikova@utb.cz (B.H.); slobodian@utb.cz (P.S.); 2 Department of Physics and Materials Engineering, Faculty of Technology, Tomas Bata University in Zlín, Vavreckova 5669, 760 01 Zlín, Czech Republic; 3 Department of Chemistry, Faculty of Technology, Tomas Bata University in Zlín, Vavreckova 5669, 760 01 Zlín, Czech Republic

**Keywords:** sensor, graphene, co-polymer, chemiresistivity, organic vapour sensing, Hansen solubility parameter

## Abstract

A styrene-butadiene-styrene co-polymer matrix nanocomposite filled with graphene nanoplatelets was studied to prepare chemiresistive volatile organic compounds (VOCs) room temperature sensors with considerable response and selectivity. Nanofiller concentration was estimated from the electrical conductivity percolation behaviour of the nanocomposite. Fabricated sensors provided selective relative responses to representative VOCs differing by orders of magnitude. Maximum observed average relative responses upon exposure to saturated vapours of the tested VOCs were ca. 23% for ethanol, 1600% for acetone, and the giant values were 9 × 10^6^% for n-heptane and 10 × 10^6^% for toluene. The insensitivity of the sensor to the direct saturated water vapour exposure was verified. Although high humidity decreases the sensor’s response, it paradoxically enhances the resolution between hydrocarbons and polar organics. The non-trivial sensing mechanism is explained using the Hansen solubility parameters (HSP), enabling a rational design of new sensors; thus, the HSP-based class of sensors is outlined.

## 1. Introduction

Graphene, discovered by A. Geim and K. Novoselov in 2004 [1], is an ideal material for gas sensing. Monolayer graphene has an extremely large specific surface area theoretically calculated up to 2630 m^2^·g^−1^ [2] and unique mechanical and electrical properties, especially remarkable electronic mobility theoretically calculated at ~200,000 cm^2^·V^−1^·s^−1^ if the extrinsic disorder is eliminated [3]. The adsorption of molecules, which can be either donors or acceptors of electrons, alters the charge carrier concentration, significantly changing the electrical resistance of the material. The extraordinary sensitivity of graphene even enables the detection of single gaseous molecules [4].

To impart selectivity and utilise the tremendous potential of graphene, variations in graphene dimensionality, doping, chemical modifications, and the preparation of hybrid nanocomposites were investigated [5]. In most cases, the additional component modifies the character of graphene to a semiconductor and imparts a specific interaction with the analyte molecules [6]. Nevertheless, the technological readiness of graphene-based gas sensors still lags behind their contemporary, more material-demanding and energy-demanding substitutes [7].

In contrast to the intriguing chemistry of the above-described gas-sensing elements, the mechanism of strain sensors and their design is much simpler. The response is based on the conductivity of a graphene filler network dispersed in a polymer matrix whose conductivity depends on percolating pathways established by contacts of the filler particles. Hopping and tunnelling are the two possible mechanisms of charge transfer. The strain response of the graphene network mainly depends on changes in the contact resistance of adjacent graphene sheets [8]. If the interparticle contact creates a narrow neck limiting the overall conductivity, the adsorption of gaseous molecules will influence the resistance response of such a material as much as it changes the quality of these contacts. Our previous work on carbon nanotube entanglements demonstrated this sensing mechanism [9,10].

Hence, a nanocomposite material could provide a response to both adsorbed gas molecules and applied strain. Nevertheless, the strain-induced resistance changes are undesirable in VOC sensors and must be minimised in preparing flexible and stretchable devices to avoid confusion of the signals with the desired response to gas molecules [11]. On the other hand, integrating both sensing functions into one material might be promising, especially in wearable electronics [12].

We hypothesise that (1) a graphene filler-based polymer matrix composite can be highly suitable for fabricating gas sensors. The polymer matrix may impart a significant sensor selectivity to various organic vapours based on the specific polymer-solvent interaction described using the Hansen solubility parameters (HSP) approach, which has surprisingly gone almost unnoticed in the available literature, except in one case. C. Rattanabut et al. developed graphene and poly(methyl methacrylate) (PMMA) composite laminates on flexible substrates for VOC detection. The addition of the polymer increased the response of the graphene/PMMA sensor to dichloromethane three times and almost entirely suppressed the response of the sensor to acetone, chloroform, and benzene compared with the graphene sensor [13,14]. This was confirmed in an electrochemical sensor [15] and also demonstrated in optical sensing [16]. Polymer swelling was also noticed as the sensing mechanism for polymer-coated carbon nanotubes. Our pioneering work demonstrated the ability of various polymer matrices to induce reasonable selectivity in carbon nanotube-filled nanocomposite layers in VOC sensors [17]. Furthermore, the sensitivity of the graphene-filled composite should be enhanced (2) by choosing its composition close to the percolation threshold. Sensitivity and selectivity maximisation work in synergy resulting in a high response of the sensor to specific stimuli. As the last part (3), the thickness of the active sensing composite layer should be optimised and thick enough to provide sufficient conductivity and homogeneity regarding the dispersion and distribution, shape, and size of the filler particles, whereas it should be as thin as possible to suppress the importance of undesirable effects of transport phenomena, diffusion, and accumulation of the adsorbed organic molecules in the layer. Following these three design rules, we prepared the first simple styrene-butadiene-styrene/graphene composite VOC room temperature sensor device, reporting here its giant relative response and selectivity to vapours of exemplary VOCs. The aim of our pioneering study is to pave the way for a new class of sensors utilising the HSP approach in the formulation of the matrix-filler composite active layer to achieve effective room temperature sensors for VOCs. Further systematic studies using standard and more elaborated sensor characterisation techniques will be necessary.

## 2. Materials and Methods

### 2.1. Materials and Sample Preparation

For the preparation of nanocomposite material, graphene nanoplatelets (GNPs) xGnP M-5 were used (Sigma Aldrich, St. Louis, MO, USA). GNPs are supplied in powder form, having a declared average particle size of 5 μm, a thickness of 6–8 nm, a specific surface area of 120 to 150 m^2^/g, and a density in the range of 0.03–0.1 g/cm^3^. As the polymer matrix, SBS (styrene-isoprene-styrene co-polymer) was chosen. SBS, product number 432,490 (Sigma Aldrich, St. Louis, MO, USA), with a 30 wt.% of styrene content, density 0.94 g/cm^3^, hardness 61 Shore A, was purchased.

Absolute ethanol, acetone, toluene, and n-heptane of analytical purity (all purchased from Sigma Aldrich, St. Louis, MO, USA) were used as representative VOCs throughout the experiments. The conductivity of the water used was 0.09 µS/cm with 6.3 pH (due to the absorption of CO_2_ from the air).

First, the stock solution of SBS in toluene (*w_t_* = 18.7%) was prepared by dissolving in toluene under constant stirring at 65 °C for 24 h. Then, GNPs were added to the SIS solution and stirred at 350 RPM for 5 min. Immediately after that, the dispersion was sonicated using the ultrasonic device UP 400 S (Hielscher, Teltow, Germany) at a pulse value of 0.5 and a sonicator power of 50% for 5 min. The obtained dispersions were used for the fabrication of SBS/GNPs nanocomposites with various nanofiller concentrations (mass percent of 1, 2.5, 3.75, 4.5, 5, 7.5, 10, 15, and 20%) for percolation curve examination. The material with 7.5% of GNPs in the SBS/GNPs nanocomposite was then selected and used to prepare the active layer in the sensor specimens.

**Figure 1:**
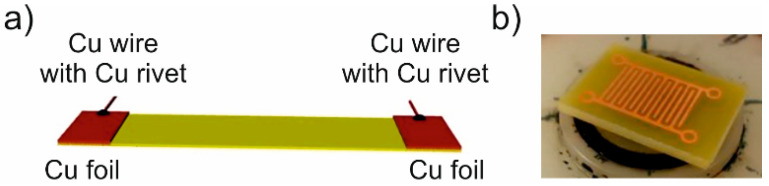
(**a**) Schematic view of a substrate with electrodes for measuring the dependence of GNPs composite electrical conductivity on filler concentration; (**b**) a photograph of an IDE substrate for VOC sensors.

Specimens for determining the percolation threshold and interdigitated electrode (IDE) sensors were made similarly to our previous works with carbon nanotubes [17,18]. Rectangular substrates (sized 50 mm × 20 mm) with two side electrodes were prepared for percolation curve measurements, whereas IDE patterned substrates (sized 20 mm × 13 mm, gap width 0.5 mm) were prepared for the fabrication of sensors as depicted in Figure 1. Subsequently, the substrates were coated with the polymer dispersion at 1500 RPM in a spin coating machine Laurell WS-650Mz-23NPP (Laurell Technologies, Lansdale, PA, USA) for 1 min to form the nanocomposite layer. Prepared specimens were then dried at 25 °C for 48 h. The ensuing films were about 8 μm to 10 μm thick. The thickness of the active composite layer was measured by optical profilometry (see below).

### 2.2. Characterisation Techniques

SEM analysis was performed using a microscope Nova NanoSEM 450 (FEI, Hillsboro, OR, USA) with an autoemission cathode, point resolution ≤ 1.0 nm at 15 kV. The samples were gold sputtered using a device Q300 TT (Quorum Technologies, Laughton, UK). Cross-sections were obtained by fracturing in liquid nitrogen. Transmission electron microscopy (TEM) of GNPs was performed with a TEM microscope JEM—2100/HR (JEOL, Tokyo, Japan) to complete the microscopic characterisation of graphene material. The thickness and roughness of the nanocomposite films were measured using a 3D optical profiler ContourGT-X (Bruker, Billerica, MA, USA).

The electrical conductivity was estimated from resistance measurements performed with a Keithley 6517B (Tektronix, Inc., Beaverton, OR, USA) instrument to obtain the percolation curve of the SBS/GNPs. Each sample was connected with the use of the two-point method.

**Figure 2:**
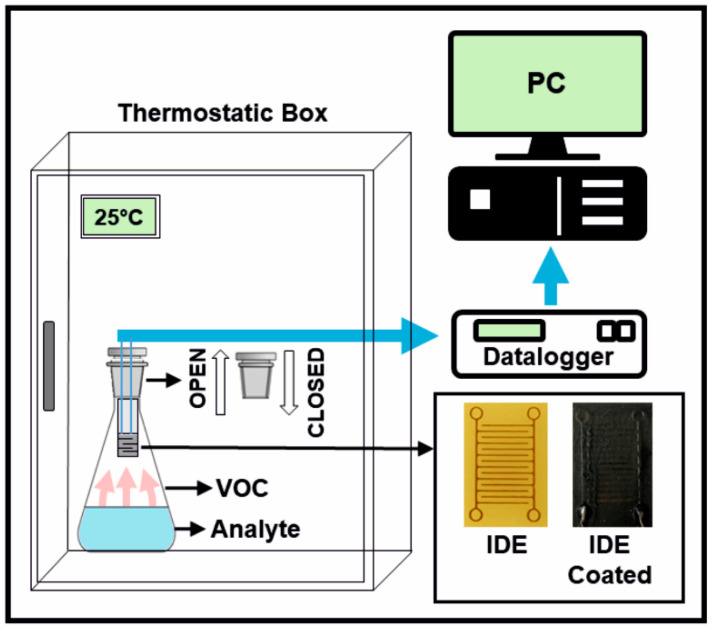
Experimental system for measuring the resistance change response to saturated vapours of the tested VOCs.

The procedure for the VOC detection and testing of the sensor response was performed using a home-built setup. The sensor was integrated into an Erlenmeyer flask plug. The exercise proceeded so that the Erlenmeyer flask containing an organic solvent was closed with a simple plug which was replaced periodically with the plug with the sensor to realise the on/off cycle, as schematically depicted in Figure 2. Further details are available in the Supplementary Material. The electrical resistance response of the sensor is manifested as the change in its resistance (∆*R*, [Ω]). The response (*R*, [Ω]) can be recorded and evaluated simply as the resistance or as a relative response (∆*R*/*R*_0_, [%]) quantified as the relative electrical resistance change:

(1) $$∆R/R_{0}=\frac{R-R_{0}}{R_{0}}\times 100\%,$$

*R*_0_ is the stabilised electrical resistance of the sensor before the exposition to vapour, and *R* is the resistance during the exposition of the holder with the IDE coated with the nanocomposite. The maximum response (*R_max_*, [Ω]) or the maximum relative response (*R_max_*/*R*_0_, [%]) of the sensor is observed at the partial pressure of saturated vapour of a given VOC at 25 °C.

The selectivity (*σ*_1/2_) is defined as the mutual property of a pair of gasses (vapours) indexed by 1 and 2, comparing their maximum relative responses normalised corresponding to the partial pressure of their saturated vapours (*p*_0_).

(2) $$\sigma_{1/2}=\frac{\left(R_{max,\;1}/R_{0}\right)}{\left(R_{max,\;2}/R_{0}\right)}\cdot \frac{p_{0,2}}{p_{0,1}},$$

Cross sensitivity towards humidity was studied using mixtures of tested liquids with water. As toluene and heptane are immiscible with water, the vapours in the Erlenmeyer flask were composed of saturated vapours of the respective VOCs and water. In the case of acetone and ethanol, mixtures of water with the respective VOCs were used to generate the gas phase of equilibrium composition.

## 3. Results and Discussion

### 3.1. Material Characterisation

**Figure 3:**
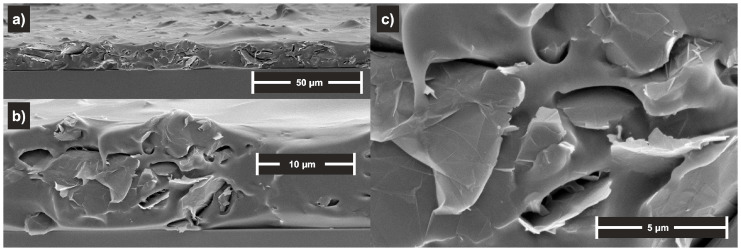
SEM images of the spin cast nanocomposite layer freeze-fracture sample at (**a**) low, (**b**) middle, and (**c**) high magnification.

The SBS polymer matrix was chosen because of its solubility, solution processability in toluene, and its ability to form elastic material consisting of two separated phases formed from the polystyrene and polybutadiene rubber blocks. GNPs were readily dispersed in SBS solution and spin cast immediately from freshly prepared dispersions. Figure 3 shows the cross-section morphology of the prepared nanocomposite as observed by SEM on the freeze-fractured sample. SEM and TEM images of GNPs are given in the Supplementary Material in Figures S1 and S2. A relatively good, although imperfect, dispersion and distribution of the GNPs filler in the SBS matrix is visible, and filler particles seem randomly oriented. The thickness corresponds with the profilometric measurements.

**Figure 4:**
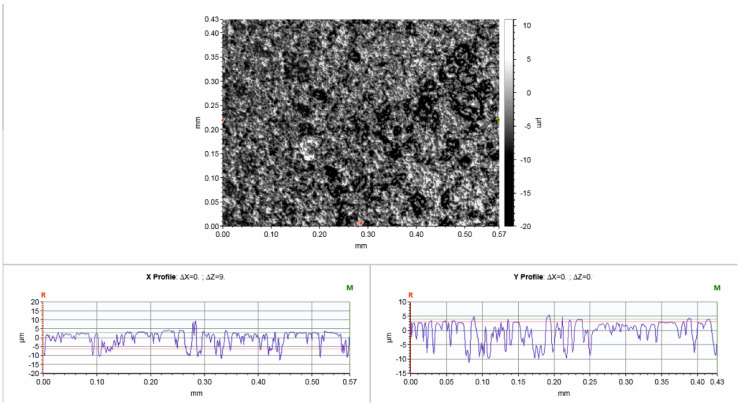
Surface topography of the SBS/GNPs nanocomposite layer from a 3D optical profiler.

A large-scale top view of the morphology of SBS/GNPs films was enabled using the 3D profiler. A rough surface on the micrometre level is observable in Figure 4. The film surface shows a relief morphology with valleys and holes ranging from shallow to deep, reaching down to the substrate. The block co-polymer forms a bi-phasic structure (polystyrene, PS, and polybutadiene, PB, phases) embedding the GNPs resulting in a granular-like surface appearance. The restacking of GNPs may also occur as a result of the destabilisation of the dispersion during drying. 

The composition of the material was confirmed by thermogravimetric analysis. The results are shown in the Supplementary Material in Figure S3. The analysis did not reveal any deviation from the initial composition indicated by the material preparation protocol. 

### 3.2. Electrical Properties of SBS/GNPs Nanocomposite Films

**Figure 5:**
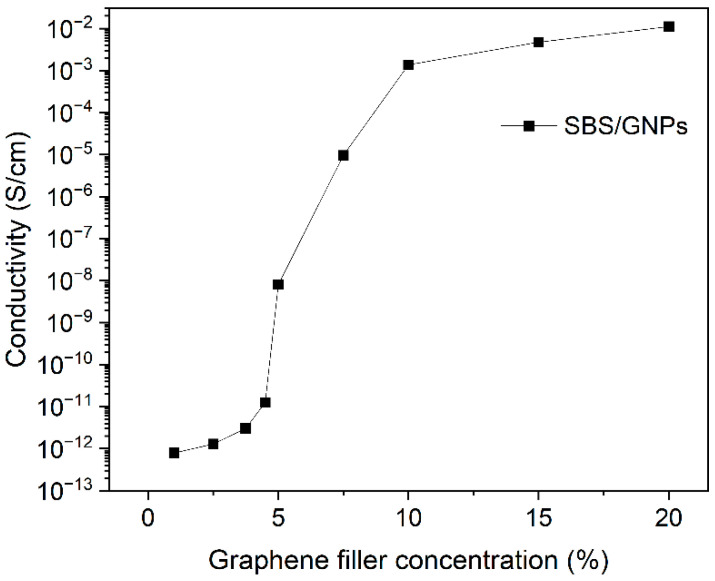
Percolation curve—Dependence of the electrical conductivity of SBS/GNPs on graphene concentrations. The lines connecting points serve only as a guide for the eye.

The resistivity of SBS/GNPs films with various filler concentrations was investigated to find a suitable concentration for preparing the active layer in the proposed sensors. The percolation curve is depicted in Figure 5. The electrical conductivity of the SBS/GNPs follows the percolation theory, indicating a percolation threshold [19]. The mechanism governing the percolation behaviour of SBS/GNPs is expected to be similar to carbon nanotube-filled composites [20]. The biggest jump by eight orders in conductivity is between 4.5% and 10%, indicating the threshold at 5% when large clusters begin to be formed and the tunnelling effect between GNPs is manifested. Above 10%, the GNPs network is formed, and a further increase in concentration only densifies its structure, manifested in incremental conductivity enhancement and following stabilisation.

Assuming a negligible difference between graphene and polymer matrix density, the value of the percolation threshold filler concentration in our SBS/GNPs is about ten times higher than the best values reported for other composites below 0.5% of volume [21,22]. This can be ascribed to the difference in the properties of various polymer matrices, fabrication methods, and GNPs used in our study and other studies. In agreement with the characterisation of the source materials and the prepared nanocomposite, we do not expect to achieve complete graphene monolayer dispersion in our samples. The threshold at a mass concentration of 5% is most likely observed due to the incomplete filler dispersion and possibly restacking of GNPs during the nanocomposite film forming.

### 3.3. Selection of the Best SBS/GNPs Composition for Sensor Design

In analogy to carbon-black-filled polymers, tunnelling or possibly hopping of charge carriers across the gaps between filler particles dictates the conductivity above the percolation threshold [23]. Let us assume a sensor as proposed in the introduction. The polymer matrix plays two roles. It fills the gaps thus controlling the tunnelling barrier, and the percolating filler network is embedded in the matrix. Interaction of the polymer matrix with suitable VOCs molecules results in the swelling and softening of the matrix. The swelling can modify the tunnelling barrier, and the softening causes relaxation and changes in the filler network, which would hardly influence the conductivity of the material below the percolation threshold. Both a high value of *R*_0_ and the highly conductive composite are unsuitable for the intended sensor function. Although the off value of *R*_0_ is small, the expected conductivity changes due to the adsorption of vapours would also be small. Therefore, the best chance for the highest sensitivity is at the threshold, but the resistance may easily exceed the measuring range of a simple electronic device. Thus, being closer to the upper conductivity level is a better strategy. It offers a reasonable *R*_0_ value and maintains a high enough slope of the percolation curve. The best point in the graph in Figure 5 seems to be at the filler mass concentration of 7.5%. It also offers a sufficiently large range of *R* to which the resistance may grow upon exposure of the sensing layer to the analyte and, therefore, was chosen for sensor fabrication in our study.

### 3.4. Response and Selectivity of the Sensor

**Figure 6:**
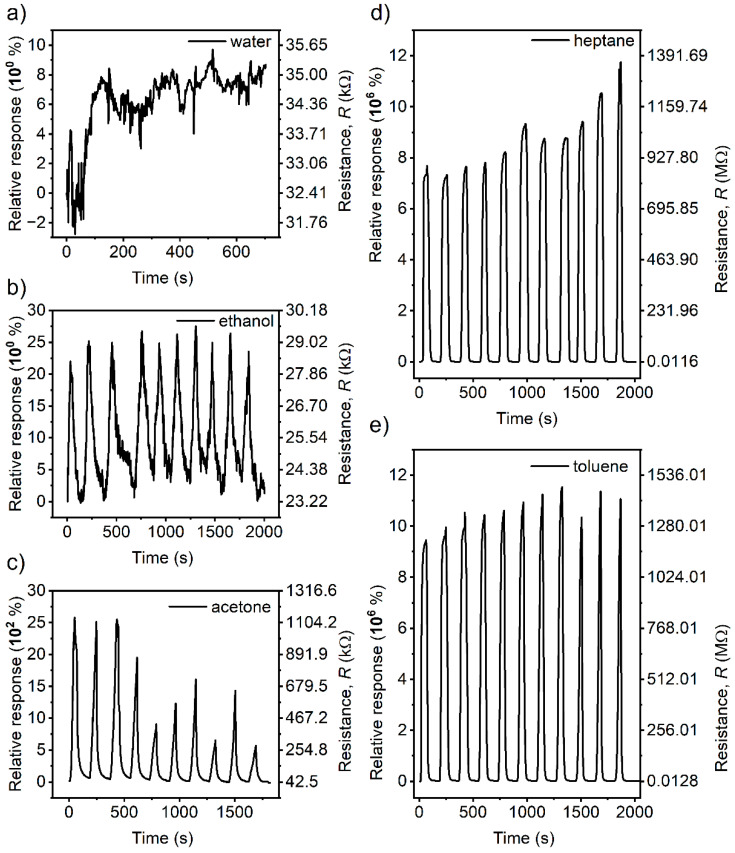
Graphs of the sensor resistance (*R*) responses and relative responses of the SBS/GNPs sensor during the absorption/desorption cyclic phase for saturated vapours of all solvents, namely (**a**) water, (**b**) ethanol, (**c**) acetone, (**d**) heptane, and (**e**) toluene. Note the *y*-scales of the graphs—the units are kΩ for water, ethanol, and acetone, whereas the graphs recorded for toluene and heptane use MΩ, as the sensor resistance changes are very large. The same scales apply for the relative response (Δ*R*/*R*_0_) expressed in % starting from units 10^0^, hundreds 10^2^, up to millions 10^6^.

The sensors were tested against vapours of water, ethanol, acetone, toluene, and heptane for ten on/off cycles as plotted in Figure 6.

The interaction of the sensor with humidity is an obligatory preliminary question of vital importance for room temperature application. Figure 6a shows the reaction of the sensor to relative humidity change from 40% in the thermostatic box to 100% in the testing flask. There was no significant response to the cycling; therefore, we concluded that water has no or negligible role in sensing in the given conditions. Then, the other VOCs were tested. The adsorption/desorption cycles yielded the typical shape of the response curves illustrating the giant relative response of sensors to some of the tested VOCs reaching up to millions of %, which in terms of electrical resistance represents a change from several tens of kΩ to several GΩ. It must be noted on account of the presented data that the testing apparatus and the method are elementary, and these measurements represent a pioneering study which will be further elaborated in more detailed research.

The observed highest *R_max_*/*R*_0_ and rounded average *R_max_*/*R*_0_ values are summarised in Table 1. The highest maximum relative response was recorded for toluene and heptane. In contrast, acetone vapours induced a small response, whereas ethanol vapours generated only small changes in the resistance. 

Table 1 also summarises the response time (*t*_90_) necessary for the sensor to reach 90% of the maximum signal in the on phase of the cycle and the recovery time (*t*_10_) necessary for the sensor to reach 10% of the preceding maximum response in the off phase of the cycle. The values were read from the graphs in Figure 6 and are presented as average values with standard deviations. The values were obtained for the tested VOCs while water had no significant response.

Table 2 summarises the selectivity (*σ*_1/2_) of the sensor towards vapours of the tested VOCs.

### 3.5. Cross-Sensitivity of VOCs to Humidity

Whenever a sensor is expected to operate at room temperature, cross-sensitivity to humidity must be investigated, as this is an omnipresent factor that cannot be avoided, as it is assured in the case of sensors operating at high temperatures. Although no significant reaction of the sensor towards saturated water vapour exposure was observed, as shown in Figure 6a, water adsorption can interfere with how the sensor reacts to the target molecules. Therefore, the sensor was exposed to mixed environments containing both the tested VOC and water vapours. Observed results are embodied in Figures S4 and S5 in the Supplementary Material.

Figure S4 shows a clear response of the sensor to a change in the environment. Toluene and heptane are immiscible with water, and it is easy to prepare a reproducible gas phase with saturated vapours of the VOC and water. First, the three on–off cycles were conducted in the flask with saturated vapours of toluene, followed by three on–off cycles in the flask with mixed saturated vapours of toluene and water (Figure S4a) and heptane and water (Figure S4b). The response of the sensor is slightly diminished to 2/3 of the value without added water due to the effect of the water vapour at the relative humidity of 100% (corresponding to a partial pressure of ca 3.17 kPa) for both hydrophobic liquids (toluene and heptane). The observed behaviour is well reproducible.

Unlike the hydrophobic VOCs, the tested polar VOCs are fully miscible with water. Therefore, the equilibrium pressure of the saturated vapours depends on the composition of the liquid phase beneath the gas phase. Mixtures of 5 vol % concentration of acetone in water and ethanol in water were used to examine the effect of the mixed VOC and water vapour on the sensor response. In this condition, neither vapour from the two reaches 100% RH. The results are shown in Figure S5a for acetone and Figure S5b for ethanol. The behaviour of the sensor in the vapours of the acetone–water mixture still carries the patterns of cyclicity and response to the change in the gas phase environment. However, the dominant water vapour component effect results in a stable signal decrease. The sensor behaviour in the ethanol–water mixture vapours showed a depreciation of the signal after exposure to the almost saturated water vapours with the small contribution of ethanol. Figure S5b shows the cycles for the sensor after being transferred from the standard procedure with the pure ethanol vapours to the vapours of the mixture. After the first cycle, the response fell to a mode of insignificancy, as observed for pure water vapours earlier.

### 3.6. Sensing Mechanism 

As presumed in the introductory part, the mechanism imparting the giant response and selectivity to the SBS/GNPs sensors can be described in terms of polymer swelling due to the adsorption of VOC molecules and their interaction with the polymer matrix. Nevertheless, some other possible mechanisms need to be reconsidered, including the interaction of the VOC molecules with the filler particle network.

First, let us consider solvent polarity as a critical factor in the simple electrostatic model of solvation, where the relative permittivity represents the ability of a solvent to separate charge and orient its dipoles [28]. Here, the molecules with higher polarity could influence the band structure of the conductive filer and interact with charge carriers, eventually holding the holes in the graphene layer and dumping their moving, resulting in a decrease in electrical conductivity [13]. The values of solvent permittivity *ε_r_* taken from the reference [28] are in the order of water 78.36, ethanol 24.55, acetone 20.56, toluene 2.38, and heptane 1.92, which shows a negative correlation with the observed responses of the sensor. The direct effect of the solvent molecule polarity on GNPs is probably too small, or it does not enter the game at all.

The second simple parameter to consider is the molar volume (*V_m_*) of the tested VOCs. According to the literature [29], the value of *V_m_* of water is 18.0 cm^3^/mol, ethanol 58.5 cm^3^/mol, acetone 74.0 cm^3^/mol, toluene 106.8 cm^3^/mol, and heptane 147.4 cm^3^/mol. A positive correlation in this series can be related to the swelling and increase in the polymer matrix volume, which may result in pushing the filler particles apart and tunnelling charge transfer depreciation. This effect seems to be included in the solubility effect discussed later. On the other hand, the diffusion of solvents into polymers might be a critical process in the sensing mechanism of the sensors in question with respect to the response and recovery time window and the duty cycle. The diffusion coefficients (*D_s_*) of solvents in rubbery polymers range from 10^−13^ m^2^/s to 10^−8^ m^2^/s [30], suggesting a possibly large influence. The diffusion is impeded by increasing the molecular volume of the solvent molecule, as log *D_s_* depends on molar mass linearly with a negative slope [31]. It can be seen in Table 1 that the response and recovery times have a negative correlation with the molecular volume. Accordingly, the molar volume does not affect the sensor response in this way.

Finally, let us discuss the SBS co-polymer matrix VOC interaction. Although empirical and lacking the effect of temperature, the Hansen solubility parameter (HSP) pragmatic approach fits well in the framework of classical polymer solution theories and has earned its recognition in the prediction of polymer solubility [29]. The dissolution and swelling of polymers are governed by the similarity of the solvent and the solute in three relevant parameters describing the effect of the dispersion force (δ_D_), the polar intermolecular force (δ_p_), and the hydrogen bond (δ_H_). These three HSP (in MPa^1/2^) describe the major contributions to cohesion energy and create a three-dimensional space where a point represents a solvent or solute. The square of the Euclidean distance, (*r*_a_)^2^, between two points in the HSP space describes the difference in cohesion energy per molar volume between these two components, which is used to assess their mutual ability to form a solution (miscibility, in other words). The empirical parameter is the interaction radius (*r*_0_) which expresses the maximum energy difference allowing the solubility of the compound in question. This radius is indeed a Euclidean distance and defines the best spherical approximation of the volume surrounding the solute point in the HSP space embracing experimentally estimated solvents, the so-called interaction sphere. It is also called a solubility sphere as it encompasses good solvents for polymers. In contrast, points lying outside the sphere shall not dissolve the solute. Some thermodynamically bad solvents or even good solvents may fall out of the sphere as the estimation of its radius is based on statistics. Nevertheless, points representing such solvents will not be far from the sphere’s surface. The relative energy difference (*RED*) used to predict the solubility of a polymer in a solvent is defined as:

(3) $$RED=\frac{r_{a}}{r_{0}}$$

(4) $${\left(r_{a}\right)}^{2}=4{\left(\delta_{D,pol}-\delta_{D,sol}\right)}^{2}+{\left(\delta_{p,pol}-\delta_{p,sol}\right)}^{2}+{\left(\delta_{H,pol}-\delta_{H,sol}\right)}^{2}$$

The lower indexes “pol” and “sol” stand for polymer and solvent, respectively. Note that the lowercase symbol “*r*” is used here for the radius instead of the commonly used symbol “*R*” to avoid confusion with the electrical resistance.

The biphasic SBS co-polymer matrix in the nanocomposite can be treated as a combination of two model polymers PS and PB. Table 3 summarises the tabulated HSP of tested VOCs and the model polymers, the interaction radii of PS and PB, and *RED* calculations. A *RED* value lower than 1 indicates the high miscibility of the VOC in the polymer in question. Moreover, the smaller the *RED* value is, the better the VOC is as a solvent for the polymer. For example, ethanol and acetone have values of *RED* higher than 1, correlating to the observed small relative response. Nevertheless, the acetone/ethanol selectivity value is 25, ascribed to the differences in their *RED* values calculated for both polymer phases. Indeed, neither PS nor PB dissolves in ethanol, whereas acetone nearly approaches the limit of good solubility defined by the value of 1 for both polymers, thus providing limited solubility for SBS. Unlike this simple picture, a more complicated pattern can be seen in the case of toluene and heptane. Toluene is the best-scoring solvent for both polymers and totally dissolves PS, PB, and the SBS co-polymer. In contrast, heptane does not dissolve PS, whereas it is a good solvent for PB. The relative response and selectivity of the sensor towards the vapours of all of the tested VOCs follow the order of the corresponding HSP values. Water is considered in Table 3 to complete the picture. Indeed, it causes no significant sensor response, which correlates with its high RED and the fact that both PS and PB are insoluble in water and do not swell. On the other hand, it must be emphasised that water is an enfant terrible in the HSP approach. The parameters for a single water molecule derived from the energy of vaporisation of water at 25 °C [29] were used in this table as this may correspond to the very low concentration of the molecules in the polymer or to the gaseous phase at best.

**Figure 7:**
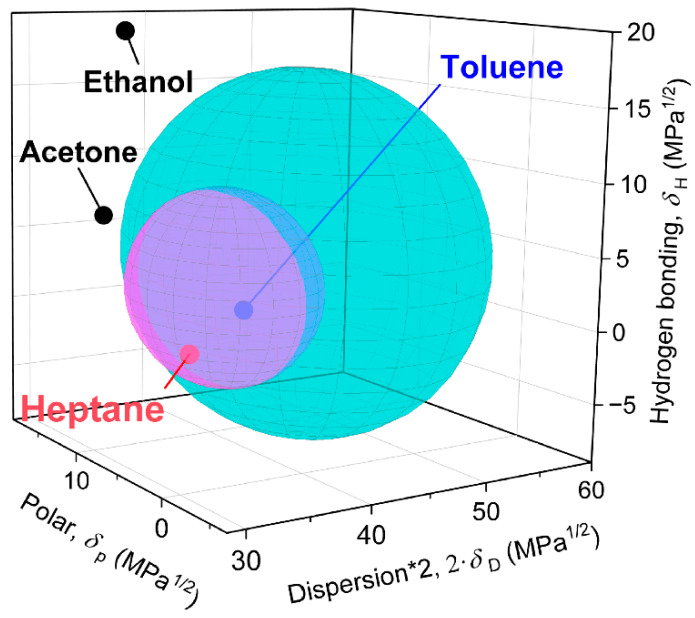
HSP plot for the tested VOCs and solubility spheres of the two components of the SBS co-polymer. Water is not plotted as this is an unnecessary outlier and would deform the scale suitable for reading the picture. Based on the data from Table 3 and references therein. The names of the VOCs are in black for those outside both spheres, red indicates belonging of the VOC to only one sphere and blue indicates belonging of the VOC to both spheres.

The HSP plot in Figure 7 offers a much more understandable picture, as it visualises the numbers in their geometrical meaning using the main advantage of the HSP approach. There are two solubility spheres, the larger in cyan, corresponding to PS with *r*_0_ value of 12.7 MPa^1/2^, and the smaller in magenta, corresponding to PB with *r*_0_ value of 6.5 MPa^1/2^.

The two spheres have an intersecting volume, defining an HSP space for the common solvents. Nevertheless, the volumes not included in the intersection are significantly larger, encompassing HSP values specific for either PS or PB components, which indicates their different solubility behaviour. Thus, the union of the two balls represents a model of the two structural components in the SBS co-polymer. The ethanol point is far from the surface, does not dissolve the polymers, and induces the sensor’s smallest response. The acetone point is outside the union volume also, but still close to both spheres and gives a much higher signal. The toluene point is inside the two spheres’ intersection and dissolves both polymer components well. The heptane point is only inside the solubility sphere of PB and is not included in the one of PS. The solubility is the main factor in the sensor’s giant response to heptane and toluene, whereas the relatively low but observable mutual heptane/toluene selectivity can be ascribed to the difference in solubility of the individual components.

It must be stressed that the used SBS block co-polymer contains only a 30 wt.% of styrene content. It is a thermoplastic elastomer with a major continuous flexible PB phase, whereas the discontinuous PS domains create rigid crosslinking points. Combining these two phases imparts rubber elasticity to the co-polymer. Toluene swells both phases of the sensor’s SBS polymer matrix well, which is responsible for the high sensitivity towards it. However, at the same time, it causes the softening, plastification, and relaxation of the whole material, including the relaxation of the GNPs filler percolating network. Therefore, the increasing distance between the filler particles is considered the primary sensing response mechanism, whereas the filler network relaxation may work in the opposite direction. Heptane does not dissolve PS, but it dissolves PB well. Therefore, the PB continuous phase becomes readily swollen when absorbing heptane vapours, while the PS domains are less affected. Both solvents efficiently swell the material, and the volume increase in the material due to swelling will induce stress rather than relaxation. This will not only depreciate the tunnelling contacts between the filler particles due to the increasing interparticle distance but the filler network can also be deformed. Such network disruptions can be especially expected at the interfaces between PB’s and PS’s phase domains due to their different degrees of swelling by the tested solvent. Moreover, the stress due to the volume expansion of the matrix can cause deformations of the GNPs sheets not only at the phase interfaces but also in the volume of phases. Bending deformations of graphene sheets will decrease their conductivity, thus contributing to the overall increase in the SBS/GNPs nanocomposite giant resistivity response when exposed to heptane and toluene vapours.

The solubility of GNPs in the used polymers and solvents must also be considered to complete the whole picture. First, it must be noted that dispersing GNPs in solvents or polymer matrices up to individual graphene sheets is not easy at all. Based on the HSP of graphene in the very authoritative reference [32], which has been estimated as δ_D_ ≈ 18.0 MPa^1/2^, δ_p_ ≈ 9.3 MPa^1/2^, and δ_H_ ≈ 7.7 MPa^1/2^, the *r*_a_ to any solvent can theoretically be estimated; nevertheless, the experimental estimation of graphene’s r_0_ remains a challenge. According to the literature, a sphere radius of 6.5 MPa^1/2^ was chosen as the best guess to differentiate between suitable and unsuitable solvents [33]; yet it can still be a matter of debate, namely the issue of acetone.

Table 4 summarises the values of the relevant parameters and results of calculations in the same fashion as Table 3. First, the interaction of graphene and polymer matrices has to be evaluated. It seems that PB is closer than PS to the surface of the graphene’s solubility sphere indicating better (yet still not good) dispersibility and, more generally, a greater affinity between the polymer and the filler than in the case of PS. The stronger interaction between PB and the graphene sheets could be due to the contribution of dispersion forces, as the δ_D_ value of PB is much closer to graphene than the δ_D_ value of PS. We also know from the previous considerations that heptane has an excellent and probably selective interaction with the PB phase. On the other hand, the *RED* value for the graphene–heptane pair suggests the worst solubility, mainly due to a large discrepancy in the effects of intermolecular forces and hydrogen bond interactions. In contrast, toluene does not provide such specificity. Thus, the co-occurrence of these two opposite mechanisms also contributes to the observed giant response and selectivity of the SBS/GNPs sensor.

The large difference between the *RED* values of ethanol and acetone in Table 4 is also worthy of attention. The difference in the affinity of acetone and ethanol to graphene may provide an additional explanation for the mechanism of the sensor’s selectivity towards these two VOCs. As it was shown, acetone is better than ethanol in swelling the co-polymer matrix components, although the poor solubility in the matrix is still the main factor limiting the sensor’s response toward both of them. Nonetheless, the *RED* of acetone to graphene is the best among all other components. As a result, the acetone molecules may preferentially be adsorbed on the graphene sheets if penetrated through the matrix. Then, they can contribute selectively to a matrix volume increase localised in the filler particles’ intimate proximity, which further impedes the tunnelling charge transport, additionally increasing the sensor’s response to acetone.

The observed effects of 100% relative humidity or nearly saturated water vapour pressure are not intrinsic. The sensor has neither a positive nor a negative response towards saturated water vapours, as shown in Figure 6a. A simple summation of the signal contributions to the overall sensor response cannot be expected. Indeed, the water does not interact with the co-polymer matrix volume regarding swelling, as discussed above. Hence, an interaction on the nanocomposite surface must be considered. It can be assumed that when the relative humidity approaches 100%, water may be adsorbed or even condensed on the rough surface of the nanocomposite, where the roughness provides large surface curvatures. Such an additional polar barrier may be responsible for the slightly diminished response towards hydrophobic VOCs, whereas its effect on the polar VOCs may be more pronounced as they are miscible with water, and local equilibria may influence the process of adsorption and desorption of the polar molecules. Whereas the signal is slightly decreased for hydrophobic molecules and remains stable, it is largely suppressed for the polar substances and shows marks of a decrease with time, which, paradoxically, contributes to the selectivity enhancement.

To emphasise finally, both of the main advantages of HSP, i.e., simplicity, explanatory and predictive power, were also proven in this study of a non-trivial graphene block co-polymer matrix nanocomposite-based sensor device, thus extending the applicability of this approach to the field of sensors. Although further systematic and more elaborated characterisation studies must be performed, we believe it is legitimate to coin the term Hansen solubility parameters-based sensors for the class of sensors working on the sensing principles demonstrated in this study which may become game-changing in the future. It is not only description and explanation that is possible now, but a rational design of new sensors of this class is enabled using the discovered pieces of the sensing mechanism and knowledge of factors playing roles in the solvent–polymer–filler interactions.

## 4. Conclusions

We succeeded in the fabrication of a chemiresistive VOC sensor using an electrically percolating SBS/GNPs nanocomposite as a sensitive layer deposited by spin coating on a copper IDE patterned substrate. Three general rules of successful material selection, material design, and sensor construction were applied. As a result, the sensor provided highly selective responses to saturated vapours of representative VOCs differing in relative response values by orders of magnitude, i.e., average maximum sensitivity (*R_max_*/*R*_0_) for ethanol 23%, acetone 1600%, up to the giant value for n-heptane of 9,000,000% and toluene 10,000,000%, which correspond to resistance response change from several tens of kΩ to several GΩ. Although high humidity somewhat decreases the response of the sensor to the tested gases at room temperature, it actually improves the selectivity of the sensor, enhancing the resolution between hydrocarbons (represented by aliphatic heptane and aromatic toluene) and polar organic compounds (represented by acetone and ethanol). 

The GNPs-filled nanocomposite material with an SBS co-polymer matrix represents a non-trivial example of graphene or any other nano-carbonaceous filler-based polymer matrix composite material for constructing chemiresitive sensors. The complex sensing mechanism of this kind of sensor is based on non-bonding intermolecular interactions. Therefore, the sensor response is governed by several effects, some of which can be synergic or antagonistic and manifested depending upon a proper sensor’s material selection, tuning electrical properties using percolation theory, and construction design. The key factors and their intriguing roles in the sensing mechanism were described and explained in terms of the Hansen solubility parameters approach, providing simple and explanatory sufficient insight into the involved intermolecular interactions. Moreover, based on the clarified sensing principles and knowing the factors playing roles in the solvent–polymer–filler interactions, a rational design of new sensors is enabled due to the HSP approach for predictive power in future. Thus, a new class of sensors is announced under the name of “Hansen solubility parameters-based” (HSP-based) sensors.

## Figures and Tables

**Table 1:** Results of maximum values Δ*R*/*R_0_* of the sensor towards the tested VOCs. The values of saturated vapour partial pressure at 25 °C are taken from the following references: (a) [24], (b) [25], (c) [26], and (d) [27].

VOC	*p*_0_ (kPa)	Maximum *R_max_*/*R*_0_ (%)	Average *R_max_*/*R*_0_ (%)	*t*_90_ (s)	*t*_10_ (s)
Ethanol ^a^	7.870	26.8	23	48 ± 13	110 ± 50
Acetone ^b^	30.868	2580	1600	52 ± 9	24 ± 4
Heptane ^c^	6.476	11,600,000	9,000,000	24 ± 5	20 ± 2
Toluene ^d^	3.8	11,500,000	10,000,000	31 ± 4	22 ± 2

**Table 2:** The selectivity (*σ*_1/2_) of the sensor towards tested VOCs.

Gas (VOC) 1		Gas (VOC) 2		
	Ethanol	Acetone	Heptane	Toluene
Ethanol	1	0.056	0.0000021	0.00000111
Acetone	18	1	0.0000373	0.0000197
Heptane	476,000	26,800	1	0.528
Toluene	901,000	50,800	1.8	1

**Table 3:** The HSP and *r*_0_ values [29], calculated *r*_a_ values, and calculated *RED* values between PS, PB, and the tested VOCs and water.

Polymers and Solvents		HSP Values				
	δ_D_ (MPa^1/2^)	δ_p_ (MPa^1/2^)	δ_H_ (MPa^1/2^)	*r*_0_ (MPa^1/2^)	*r*_a_ (MPa^1/2^)	*RED* (MPa^1/2^)
PS	22.3	5.8	4.3	12.7	-	-
Water	15.5	16.0	42.3	-	41.63	3.28
Ethanol	15.8	8.8	19.4	-	20.15	1.59
Acetone	15.5	10.4	7.0	-	14.61	1.15
Toluene	18.0	1.4	2.0	-	9.93	0.78
Heptane	15.3	0	0	-	15.75	1.24
PB	17.5	2.3	3.4	6.5	-	-
Water	15.5	16.0	42.3	-	41.44	6.38
Ethanol	15.8	8.8	19.4	-	17.6	2.71
Acetone	15.5	10.4	7	-	9.72	1.5
Toluene	18	1.4	2	-	1.94	0.3
Heptane	15.3	0	0	-	6.02	0.93

**Table 4:** The HSP and *r*_0_ values [29,32,33], calculated *r*_a_ values, and *RED* values between graphene and PS, PB, and the tested VOCs.

Polymers and Solvents		HSP Values				
	δ_D_ (MPa^1/2^)	δ_p_ (MPa^1/2^)	δ_H_ (MPa^1/2^)	*r*_0_ (MPa^1/2^)	*r*_a_ (MPa^1/2^)	*RED* (MPa^1/2^)
Graphene	18.0	9.3	7.7	6.5	-	-
PS	22.3	5.8	4.3	-	9.89	1.52
PB	17.5	2.3	3.4	-	8.28	1.27
Ethanol	15.8	8.8	19.4	-	12.51	1.92
Acetone	15.5	10.4	7.0	-	5.17	0.80
Toluene	18.0	1.4	2.0	-	9.74	1.50
Heptane	15.3	0	0	-	13.23	2.04

## Data Availability

The data are available from the corresponding authors upon a reasonable request.

## Supplementary Materials

The following supporting information can be downloaded at: https://www.mdpi.com/article/10.3390/polym16030309/s1; 1. Testing of the SBS/GNPs sensor—details on the measurement procedure; 2. SEM and TEM microscopy of GNPs—images of graphene nanoplatelets filler, including Figures S1 and S2, Figure S1: SEM micrographs of GNPs powder at lower (a) and higher (b) magnification, Figure S2: TEM micrograph of an individual pack of graphene nanoplatelets; 3. Figure S3: Thermogravimetric curves of pristine SBS, graphene (GNPs) and prepared nanocomposite layer with 7.5% of the filler; 4. Figure S4: (a) Graph above—sensor response to toluene (first three cycles) and toluene–water vapours (last three cycles); (b) graph below—sensor response to heptane (first three cycles) and heptane–water vapours (last three cycles); 5. Figure S5: (a) Graph above—sensor response to acetone (first three cycles) and acetone–water vapours (last three cycles); (b) graph below—sensor response ethanol–water vapours (showing vanishing response during six cycles).
