# Supplementary material for: Giant Response and Selectivity of Hansen Solubility Parameters-Based Graphene-SBS Co-Polymer Matrix Composite Room Temperature Sensor to Organic Vapours
Source: Polymers (Basel). 2024 Jan 23;16(3):309. doi: 10.3390/polym16030309 (PMC10857537; doi:10.3390/polym16030309)
Supplement: Supplementary file 1 [file polymers-16-00309-s001.zip › polymers-2805949-supplementary.pdf]

Supplementary material

Giant response and selectivity of Hansen solubility parameters-based graphene-SBS co-polymer matrix composite room temperature sensor to organic vapours

Rostislav Slobodian[1,*], Robert Olejnik[1], David J. Dmonte[1], Jakub Sevcik[1], Jiri Matyas[1], Marek Jurca[1], R. Blessy Pricilla[1], Barbora Hanulikova[1], Petr Slobodian[1,2], Ivo Kuritka[1,3,*]

[1]Centre of Polymer Systems, University Institute, Tomas Bata University in Zlín, trida Tomase Bati 5678, Zlín 760 01, Czech Republic

[2]Department of Physics and Materials Engineering, Faculty of Technology, Tomas Bata University in Zlín, Vavreckova 5669, 760 01 Zlín, Czech Republic

[3]Department of Chemistry, Faculty of Technology, Tomas Bata University in Zlín, Vavreckova 5669, 760 01 Zlín, Czech Republic

*Corresponding Authors

Rostislav Slobodian E-mail: rslobodian@utb.cz

Ivo Kuritka E-mail: kuritka@utb.cz

Supplementary materials contents:

1. Testing of the SBS/GNPs sensor – details on the measurement procedure

2. SEM and TEM microscopy of GNPs – images of graphene nanoplatelets filler, including Figures S1 and S2
   Figure S1: SEM micrographs of GNPs powder at lower a) and higher b) magnification.
   Figure S2: TEM micrograph of an individual pack of graphene nanoplatelets.

3. Figure S3: Thermogravimetric curves of pristine SBS, graphene (GNPs) and prepared nanocomposite layer with 7.5 % of the filler.

4. Figure S3: a) Graph above - sensor response to toluene (first three cycles) and toluene-water vapours (last three cycles); b) graph below - sensor response to heptane (first three cycles) and heptane-water vapours (last three cycles).

5. Figure S4: a) Graph above - sensor response to acetone (first three cycles) and acetone-water vapours (last three cycles); b) graph below - sensor response ethanol-water vapours (showing vanishing response during six cycles).

1. Testing of the SBS/GNPs sensor

The response of the sensor to the vapours of water, ethanol, acetone, toluene and heptane was investigated. Initially, the sensor was equilibrated to constant resistance. The sensor was introduced to the saturated vapours for 1 min (the on-phase of the cycle). Then the sensor was pulled out of the flask (the off-phase of the cycle) for 2 min. These absorption/desorption cycles were repeated ten times. Only three cycles were repeated in case of water as there was no observable response. The measurement was made in the thermostatic box under atmospheric pressure at 25 °C and relative humidity of 40 %. In the case of water, ethanol and acetone, the response of the sensor was such that the measurement of electrical resistance was carried out using Multiplexed Data Logger 34980A (Keysight Technologies, Canada), which was connected to a PC, where the measured resistivity data was read every 1 s. On the other hand, the response of the sensor to toluene and heptane vapours was in such a high resistance range that a more sensitive and precise instrument Concept 40 (Novocontrol, Germany) was used.

2. SEM and TEM microscopy of GNPs

The morphology and size of particles in the material as obtained were verified by SEM. Large agglomerates can be observed in the images in Figure S1. Sonication in toluene allowed dispersion of the material into small, few-layer thick stacks of graphene sheets corresponding to the declared thickness of 8 nm, as exemplified in TEM micrograph in Figure S2.

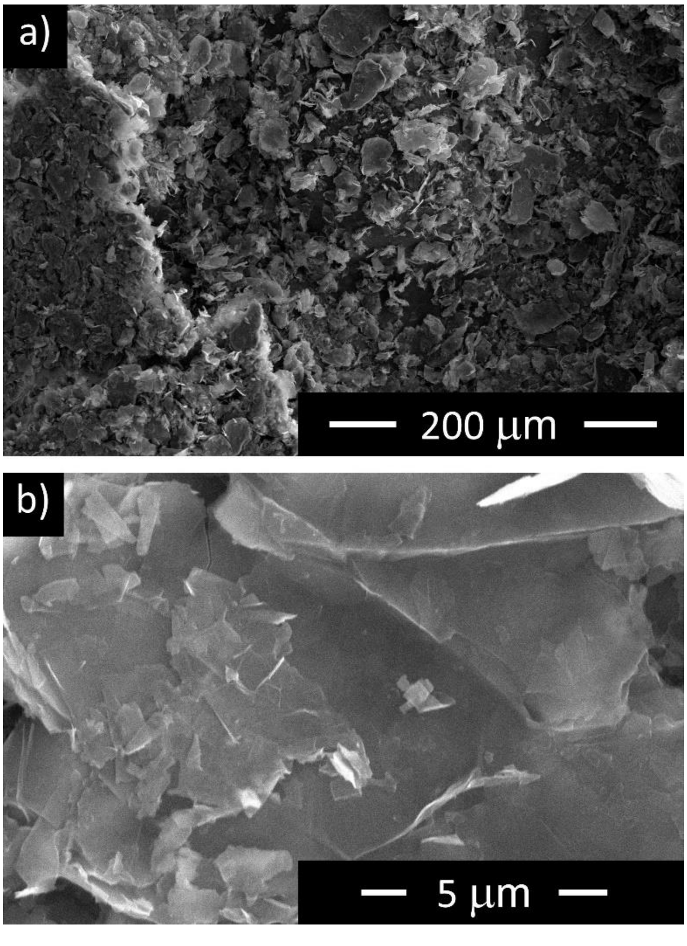

Figure S1: SEM micrographs of GNPs powder at lower (a) and higher (b) magnification.

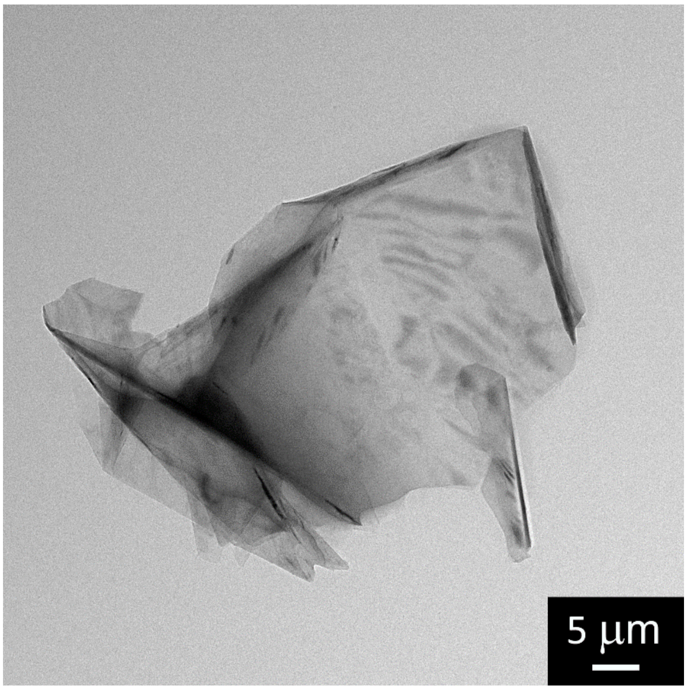

Figure S2: TEM micrograph of an individual pack of graphene nanoplatelets.

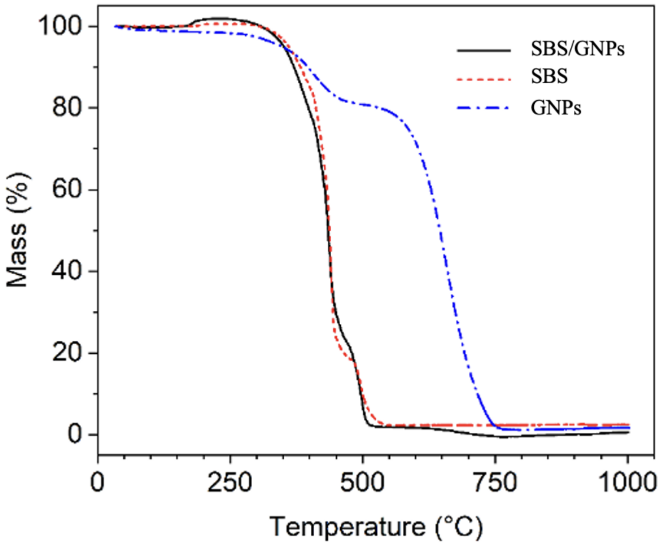

Figure S3: Thermogravimetric curves of pristine SBS, graphene (GNPs) and prepared nanocomposite layer with 7.5 % of the filler.

Thermogravimetric analysis (TGA) of source materials and prepared nanocomposite (SBS polymer, SBS/GNPs nanocomposite, and pure graphene) was performed using Setaram LabSys Evo instrument (France) with TG/DSC sensor in an air atmosphere (heating ramp 5 °C min^{-1}, up to 1000 °C, air flow rate was 60 $cm^3.min^{-1}$). The mass of the samples was 10 mg. The baseline was not subtracted.

a)

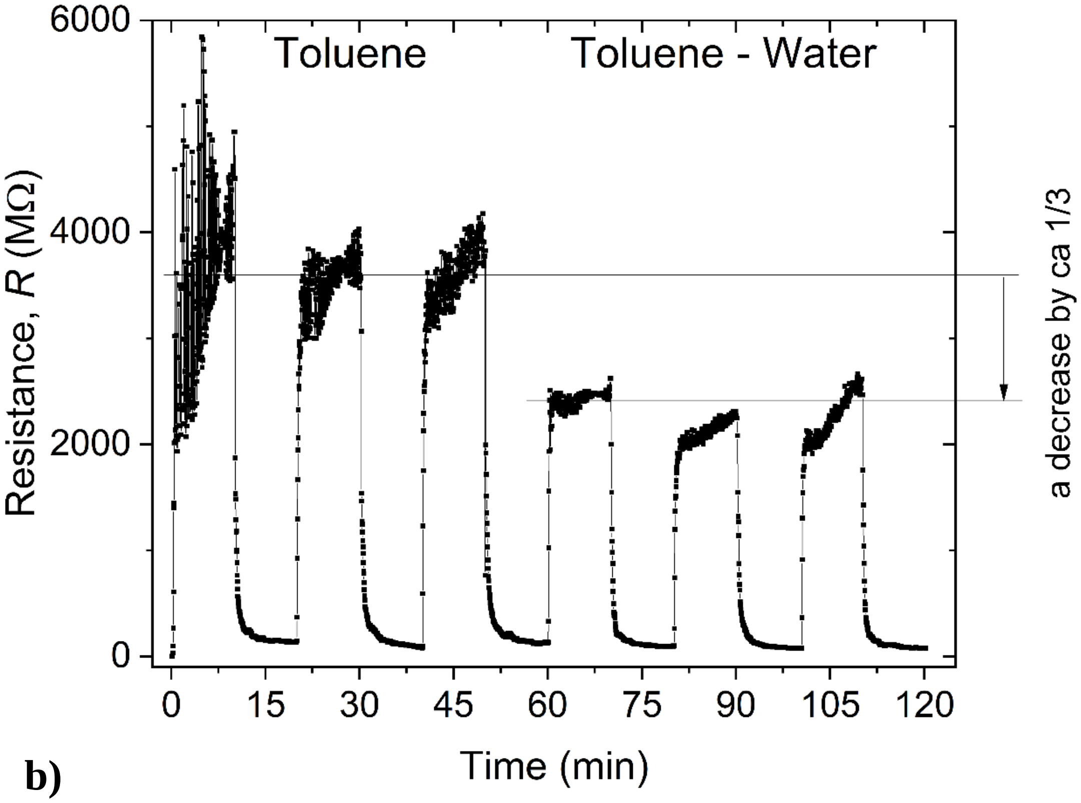

b)

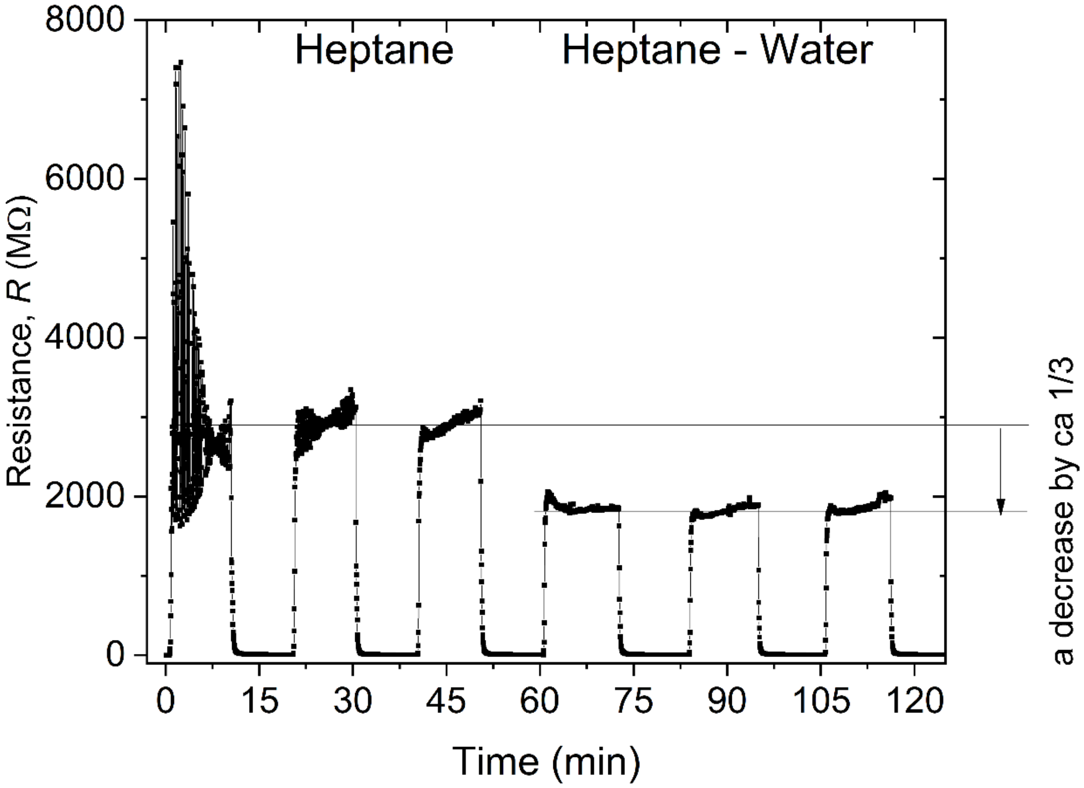

Figure S4: a) Graph above - sensor response to toluene (fist three cycles) and toluene-water vapours (last three cycles); b) graph below - sensor response to heptane (fist three cycles) and heptane-water vapours (last three cycles).

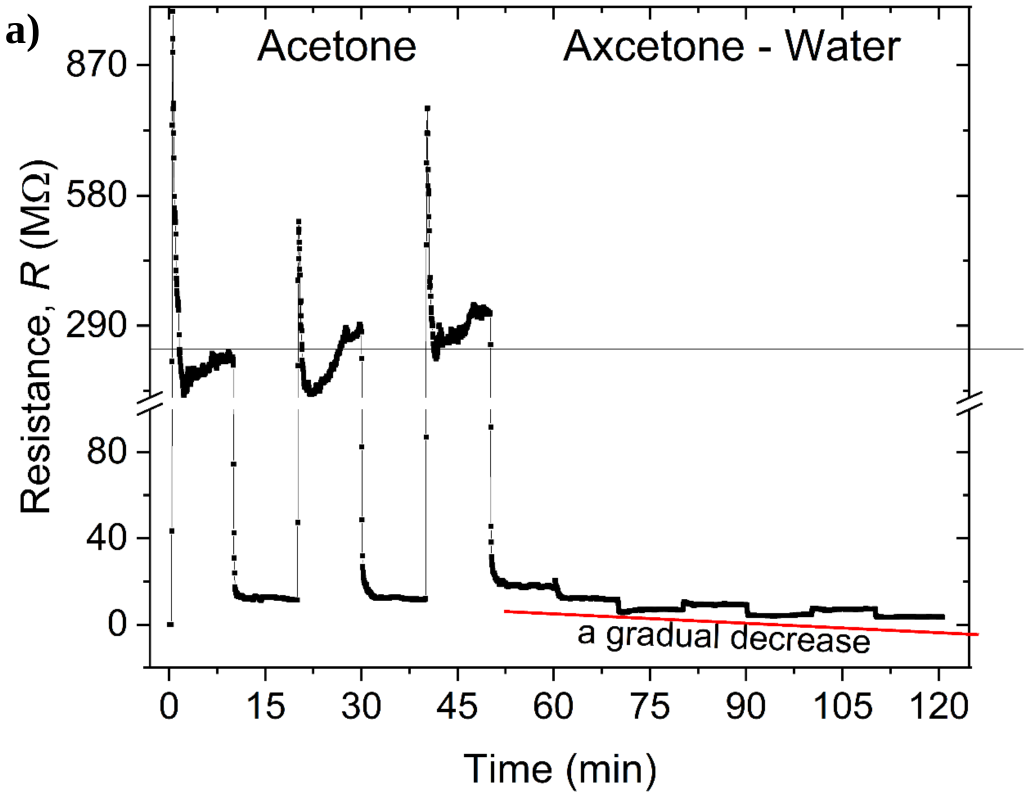

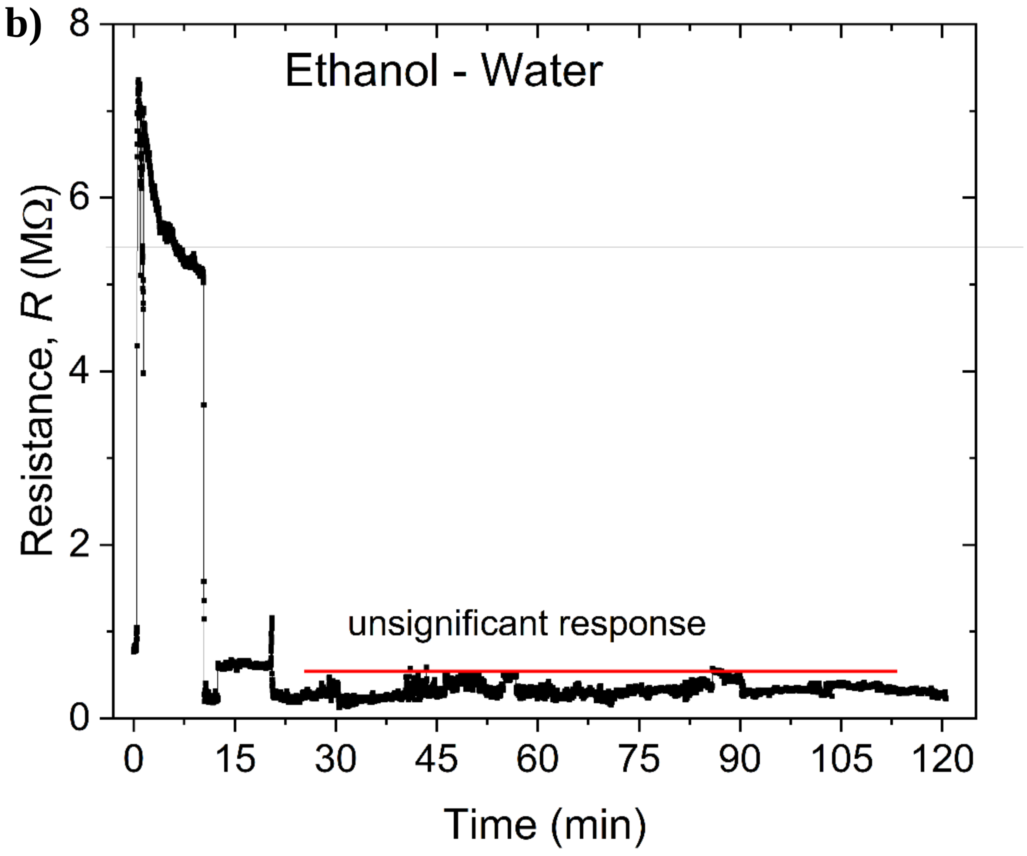

Figure S5: a) Graph above - sensor response to acetone (fist three cycles) and acetone-water vapours (last three cycles); b) graph below - sensor response ethanol-water vapours (showing vanishing response during six cycles).
